# INvestigational Vertebroplasty Efficacy and Safety Trial (INVEST): a randomized controlled trial of percutaneous vertebroplasty

**DOI:** 10.1186/1471-2474-8-126

**Published:** 2007-12-20

**Authors:** Leigh A Gray, Jeffrey G Jarvik, Patrick J Heagerty, William Hollingworth, Lydia Stout, Bryan A Comstock, Judith A Turner, David F Kallmes

**Affiliations:** 1 Department of Radiology, Mayo Clinic College of Medicine, Rochester, MN, USA; 2 Data Coordinating Center, Comparative Effectiveness, Cost and Outcomes Research Center, Department of Radiology, University of Washington, Seattle, WA, USA; 3 Department of Radiology, University of Washington, Seattle, WA, USA; 4 Department of Social Medicine, University of Bristol, Bristol, UK; 5 Department of Psychiatry, University of Washington, Seattle, WA, USA

## Abstract

**Background:**

The treatment of painful osteoporotic vertebral compression fractures has historically been limited to several weeks of bed rest, anti-inflammatory and analgesic medications, calcitonin injections, or external bracing. Percutaneous vertebroplasty (the injection of bone cement into the fractured vertebral body) is a relatively new procedure used to treat these fractures. There is increasing interest to examine the efficacy and safety of percutaneous vertebroplasty and to study the possibility of a placebo effect or whether the pain relief is from local anesthetics placed directly on the bone during the vertebroplasty procedure.

**Methods/Designs:**

Our goal is to test the hypothesis that patients with painful osteoporotic vertebral compression fractures who undergo vertebroplasty have less disability and pain at 1 month than patients who undergo a control intervention. The control intervention is placement of local anesthesia near the fracture, without placement of cement. One hundred sixty-six patients with painful osteoporotic vertebral compression fractures will be recruited over 5 years from US and foreign sites performing the vertebroplasty procedure. We will exclude patients with malignant tumor deposit (multiple myeloma), tumor mass or tumor extension into the epidural space at the level of the fracture.

We will randomly assign participants to receive either vertebroplasty or the control intervention.

Subjects will complete a battery of validated, standardized measures of pain, functional disability, and health related quality of life at baseline and at post-randomization time points (days 1, 2, 3, and 14, and months 1, 3, 6, and 12). Both subjects and research interviewers performing the follow-up assessments will be blinded to the randomization assignment. Subjects will have a clinic visit at months 1 and 12. Spine X-rays will be obtained at the end of the study (month 12) to determine subsequent fracture rates. Our co-primary outcomes are the modified Roland score and pain numerical rating scale at 1 month.

**Discussion:**

Although extensively utilized throughout North America for palliation of pain, vertebroplasty still has not undergone rigorous study. The study outlined above represents the first randomized, controlled study that can account for a placebo effect in the setting of vertebroplasty.

**Trial Registration:**

Current Controlled Trials ISRCTN81871888

## Background

Percutaneous vertebroplasty is the injection of polymethylmethacrylate (PMMA) bone cement into a painful vertebra, with the intention of providing stability and subsequently relieving pain and restoring mobility. Deramond and coworkers performed the first percutaneous vertebroplasty in France in 1984, and reported success in treating an aggressive vertebral hemangioma [1]. Soon thereafter, vertebroplasty was also used for vertebral compression fracture due to osteoporosis and tumor invasion [1].

In the early 1990's, physicians in the United States (U.S.) became interested in the use of percutaneous vertebroplasty principally for osteoporotic vertebral compression fracture. Such fractures have an incidence of approximately 700,000/year in the U.S., affecting 25% of postmenopausal women [2,3]. They may produce severe pain, as well as an increased risk of death [4-6]. Although the pain of an acute fracture is usually relieved within several weeks by bed rest, anti-inflammatory and analgesic medications, calcitonin injections, or external bracing, it can occasionally remain so severe that quality of life can be maintained only with a narcotic analgesic medication [4-6]. Moreover, prolonged immobilization caused by a fracture can have harmful effects, such as acceleration of bone resorption with increased risk for a new fracture, pneumonia, deep venous thrombosis, and decubitus ulcers [7].

## Methods/Design

### Participants and Setting

INVEST (INvestigational Vertebroplasty Efficacy and Safety Trial) is a multi-center, randomized, controlled trial comparing percutaneous vertebroplasty to a control intervention for patients with painful osteoporotic vertebral compression fractures refractory to medical therapy. The control intervention involves injection of a long-acting, local anesthetic agent adjacent to but not within the vertebral body, and no injection of cement. Subjects will be recruited from patients scheduled for vertebroplasty evaluations at participating US and foreign INVEST clinical sites. Each INVEST site will be approved by their local Institutional Review Board (IRB). Patients meeting the inclusion criteria may enroll in the study following an informed consent process.

Subjects must have a recent, painful vertebral compression fracture at levels T4 to L5 confirmed with a physical examination and radiographic imaging. The fracture must have occurred within the previous 12 months, they must have tried medical therapy for pain and have a current subjective pain rating of at least 3 on a 0 – 10 scale. Subjects are required to have a confirmed diagnosis of osteoporosis or osteopenia. Patients are excluded if they have malignant tumor deposit (multiple myeloma), tumor mass or tumor extension into the epidural space at the level of the fracture, malignancy, pedicle fracture, or cord compression. Other eligibility criteria are: at least 50 years old, no recent surgery (within 60 days), access to a telephone, no local or systemic infection, no concomitant hip fracture, no contraindications to conscious sedation, speaks English well enough to answer all health questions, not pregnant, and no evidence of dementia (able to give informed consent).

### Baseline assessment

All subjects will complete baseline assessments prior to the randomized treatment assignment. Subjects will provide the study with spine images (either plain film X-ray or MRI) to document the fracture(s). The research coordinator will complete a chart review to obtain pertinent health variables, including height and weight, prior osteoporotic fractures and back procedures, osteoporosis history, current and historical osteoporosis therapy and oral corticosteroid use, and current medication for back pain. Subjects will complete a battery of standardized measures of pain, physical disability and health related quality of life, as well as comorbidities, back pain medications and demographics. A brief physical examination will be performed, including blood pressure and brief neurological assessment (motor system, sensation, reflexes, and straight leg raises).

### Random Allocation

We will use a blocked randomization scheme with variable block sizes to ensure that the number of subjects in each treatment group (percutaneous vertebroplasty vs. control intervention) balances over time. We chose even block sizes between 4 and 12 to minimize the possibility that clinical staff will be able to guess the next treatment allocation. Randomization will be stratified by enrolment site. Random treatment assignments will be computer-generated and delivered to sites in opaque, sealed envelopes. The assignment will be revealed to the clinician in the procedure room after the subject has been sedated and has received local anesthesia.

### Procedures

Both procedures will use full sterile preparation and be placed prone on the angiographic table and receive conscious sedation by intravenous (IV) administration of fentanyl and versed and/or other medications according to the treating physician's usual practice. The subjects will have their electrocardiogram (ECG), blood pressure, heart rate and pulse oximetry continuously monitored throughout the procedure in both treatment arms. Lidocaine will be placed subcutaneously to numb the skin. Standard fluoroscopy will be used for localization of the vertebral body to be treated. The periosteum will be infiltrated with Bupivacaine using fluoroscopic guidance. In both treatment arms a small incision will be made in the skin in the vicinity of the pedicle to be treated. In the vertebroplasty arm after placement of the access needle, the cement will be mixed. The stylet will be removed leaving the cannula in place for the injection of the PMMA. In the control arm verbal clues will be given commenting on needle placement and/or cement injection with manual palpation of the area of the fracture to simulate needle placement. To further simulate the vertebroplasty procedure a monomer will be opened for the characteristic smell. Following the procedure, all subjects will be observed in the full-supine position for at least one hour. All subjects will be evaluated prior to discharge for pain, new neurological dysfunction, or other potential complications of the procedure. All subjects will be given the same instructions regarding the bandages applied to the injection/incision site and will be instructed to notify the physician with any problems.

### Crossover

If a subject has not improved by the one-month clinic visit, a crossover to the other study intervention will be offered. If the subject was randomized to vertebroplasty, the crossover procedure will be the control intervention (injection of a long-acting, local anesthetic agent). If the subject was randomized to the control intervention, the crossover procedure will be vertebroplasty. Subjects and interviewers will remain blinded to treatment assignment throughout the study, regardless of whether a crossover occurs. In the case of a crossover, they will be blinded to the order of the treatment assignments, even though they will know that the subject has received both interventions. Follow-up for crossover subjects will continue with the schedule determined at the original randomization procedure: crossover procedures have no impact on the follow-up schedule. However, the one-month follow-up examination and interviews must be completed prior to the crossover procedure. The subject may crossover to the other procedure at any time during the study.

The study will fund the full cost of the control intervention. Each site will be asked to delay the billing to the subjects in an attempt to keep them blinded as to the procedure they receive. The costs of the vertebroplasty will be billed to the subject's insurance after the one-month evaluation. Masking of the clinicians is not possible given the nature of the interventions. The evaluators (the persons conducting the follow-up interviews) will also remain blinded to the treatment allocation. Every effort will be made to maintain naiveness of the subjects and evaluators during the course of the study.

All study participants will be assessed with in-person interviews at baseline (prior to receiving the randomized treatment) and one month, and one year. All other assessments will be via telephone interviews.

### Measures

Our primary outcomes are the modified Roland Scale [8] (a measure of back pain-related physical disability) and a 0–10 numerical rating scale of pain intensity (0 = no pain, 10 = pain as bad as could be) [9]. A 2 or 3-point difference on the Roland Scale is the smallest clinically important difference found in studies of patients with other types of back pain [8,10].

Subjects are asked about their use of medications (anti-osteoporosis, analgesic, and steroid) to assess for potential confounding in the outcomes analyses. We will also collect a self-report version of the Charlson comorbidity index [11]. Secondary outcome measures include the modified Deyo-Patrick Pain Frequency and Bothersomeness Scale [12], the SF-36 version 2 (a general health-related quality of life measure) [13], the Strength of Function (SOF) and Activities of Daily Living (ADL), the EQ-5D (a measure of health state preferences) [14], and the Osteoporosis Assessment Questionnaire (OPAQ) body image domain (an osteoporosis-specific functional status instrument) [15].

A back pain resource use questionnaire is used to assess the medical care costs associated with vertebroplasty. Subjects are asked to provide information concerning outpatient and office visits, including the medical specialty of the provider; the number of visits; the tests, treatments, or devices received at the visit; and the average length of time for each visit. Hospitalizations and ambulatory surgery are also recorded in the questionnaire. Finally, we will assess the name, dose, and frequency of use of osteoporosis medications, analgesics, and any other drugs potentially used for back pain.

Adverse events and serious adverse events will be collected at each time point (clinic visit and telephone call). Data acquired at the visits and telephone calls is entered into the electronic database. Any medical problems are "caught" in the electronic system and an automatic e-mail goes to the study principal investigator and the database controller. This facilitates quick action on any serious adverse event. The DSMB and NIAMS are notified of adverse events using KAI, Research Inc. as the intermediary for notification.

Follow up radiographs will be completed for all subjects at the 12 month clinical visit. The study will pay for these x-rays. Adjacent fractures, if there are any, will be able to be seen on these radiographs.

We will screen each subject in the intervention group and the cross-over subjects for implant-related, particulate-induced granulomatous inflammation associated with extensive localized bone resorption surrounding the implant as well as vertebral fractures adjacent to treated levels. The rates of these occurrences will be reported at the completion of the study. Osteolytic responses in the presence of an implant are not uncommon and may not induce problematic levels of bone resorption. Extensive bone resorption most likely would manifest itself in the presence of symptomatic complications. Furthermore, secondary or adjacent fractures can occur as a result of the natural progression of the underlying disease and may not be treatment related.

Each subject will answer questions about medical treatment resource use at the telephone follow up calls and at the clinic visits. This will be used for the cost analysis portion of the study. Chart review will also be used to help complete the cost analysis and drug usage of the subject while participating in the study.

### Analysis

Vertebroplasty will be considered successful if the following criteria are met at 12 months post-randomization: a 50% improvement in pain related to treated fracture(s) relative to baseline, a two-point improvement from baseline on the Roland Scale, maintenance or improvement in postoperative neurologic status as compared to preoperative neurologic status (neurologic criteria for success include lack of worsening of focal deficits or of radicular pain, weakness, or numbness), absence of serious adverse events that are device/treatment related (including secondary interventions addressing symptomatic events), and absence of new fracture at treated level(s).

The control group success criteria differ slightly from that of the treatment group because of the differences in treatment and the presence/absence of a permanent implant. The control group will be considered successful if the following criteria are met: a 50% improvement in pain related to "treated" fracture(s) from baseline measurement at one month, a two-point improvement from baseline on the Roland Scale, maintenance or improvement in postoperative neurologic status as compared to preoperative neurologic status, absence of surgical intervention or need for alternative to conservative medical therapy for "treated" fracture, resolution of fracture at "treated" level through conservative medical therapy.

We will assess the baseline equivalence of the vertebroplasty and control groups by comparing them in terms of demographic characteristics, clinical findings, symptoms, and functional status. We will similarly compare subjects enrolled in the study sites. The distributions of the study outcome variables will be examined, and may be transformed, as necessary, prior to parametric tests or multivariate analyses.

For primary analyses, an "intention-to-treat" strategy will be employed, such that each subject is analyzed in the group to which he or she is randomized, regardless of actual compliance with the intended intervention. This strategy requires minimal assumptions and maintains the random allocation, although intervention effects may be "diluted" if cross over between procedures is high. We will stratify all analyses for site. Non-adherence and/or dropout complicate analysis of follow-up data. Consideration of missing data is essential to characterize the potential for selection bias through attrition. When appropriate, missing follow-up data for subjects who withdraw will be imputed.

The primary outcomes of Roland and pain measure will be assessed at the one-month time point. This allows adequate time for any intervention benefit to manifest, and is the final assessment prior to the opportunity to select further treatment (crossover). The primary analysis will test for the efficacy of vertebroplasty using a permutation test based on the randomization distribution. Such analysis allows formal inference relying solely on the random assignment of subjects for validity (i.e., makes no additional statistical assumptions). Confidence intervals for the magnitude of treatment effect will be obtained using linear regression with a treatment indicator as the predictor of interest and using dummy variables to stratify on site. We will perform a similar analysis using the 0–10 numerical pain rating scale at one month. Secondary analyses will describe the complete time profile for the Roland and the pain ratings through one year using methods appropriate for the analysis of repeated measures [16].

In addition to hypothesis tests based on the randomization scheme, we will conduct secondary analyses that adjust for important baseline characteristics, such as baseline pain severity, and functional status. We will conduct limited exploratory analyses to assess evidence for differential treatment response by subject subgroups by testing treatment-covariate interactions for factors that are specified *a priori *in a detailed analysis plan to be created prior to data analysis. We will also perform a subgroup analysis examining whether enrolling early or late in the study had an effect on outcome. This will help determine if a significant learning curve was present and, if so, the magnitude of the effect.

Stratified 2-group methods will compare the intervention groups on the secondary outcomes at 1-month, and longitudinal analysis using linear mixed models will be used to characterize and compare the time course for the intervention groups. In addition, we will compare the two groups controlling for baseline covariates including study site, demographics, clinical characteristics, and co-morbidities such as including medical and psychological factors.

We will test the hypothesis that vertebroplasty is a cost-effective procedure for reducing pain and dysfunction due to osteoporotic vertebral fractures. We will calculate the costs to health care providers of care in the first year following randomization. We believe that the 12-month time-frame will allow adequate measurement of the important differences in cost and outcome between treatment arms. However, if significant differences persist at one year, we will construct a decision model to extrapolate the results. As an exploratory analysis, we will build a worst-case scenario model that assumes that any benefits of vertebroplasty fall off rapidly over time such that after 5 years benefits are equal between groups. Survival will be based on published studies of mortality amongst an osteoporotic population [17,18]. All costs and benefits beyond one year will be discounted at 3%, according to U.S. guidelines [18].

We will combine utilization information recorded in the resource use questionnaire with unit cost information available for drugs [19], office visits [20], office-based tests and procedures, and hospitalizations [21]. The total cost will exclude the cost of the control procedure, both for subjects initially randomized to this procedure and those who later cross over to the control procedure. The costs of the radiographs necessitated by the study protocol at 12 months post-randomization will also be excluded. Costs to healthcare payers will be combined with the out-of-pocket cost to patients of obtaining health care to calculate the total incremental cost to society of vertebroplasty. All costs will be adjusted to a common year using the medical care component of the consumer price index [18].

The primary economic outcome measure is the EQ-5D preference score [22]. The EQ-5D assesses three levels of severity (no problems, moderate problems, and extreme problems) across each of five dimensions of health (mobility, self-care, usual activities, pain/discomfort, and anxiety/depression). Responses to the EQ-5D can be converted into a weighted health state index using predetermined values derived from general population surveys [22]. This index is anchored at zero (very severe health status equivalent to death) and one (best imaginable health status). By combining this quality of life index with information on patient survival, a quality adjusted life year (QALY) can be calculated.

We will calculate the incremental net benefit (INB) statistic of vertebroplasty, defined as:

INB = λ(Effect_vertebroplasy _- Effect_control_) - (Cost_vertebroplasty _- Cost_control_),

where λ is the amount that society is willing to pay for an average improvement of one quality adjusted life year. Rather than use one arbitrary cut-off (e.g., $100,000 per QALY) to define cost-effectiveness, we will present the results as a cost-effectiveness acceptability curve (CEAC). The CEAC uses non-parametric bootstrap methods [16] to determine, for any given willingness-to-pay cut-off, the probability that vertebroplasty is cost-effective. CEAC curves enable decision-makers with different budget constraints to judge the cost-effectiveness of interventions in their setting.

In the primary analysis, we will exclude subjects with missing cost or effectiveness data. However, as data may not be missing at random, we will conduct a secondary analysis using multiple imputation methods to estimate missing data [23]. We will use univariate and multivariate sensitivity analysis to estimate which non-stochastic parameters (e.g. unit costs, discount rate) have the greatest influence on our findings.

The serious adverse event (SAE) complication rates occurring within one month of surgery for the device-treated and control subjects will be evaluated descriptively in aggregate and separately. The SAE rate for each group will be estimated and the corresponding 95% exact binomial confidence intervals will be computed. If the number of events is sufficiently large to allow more sophisticated analysis, the rates between the vertebroplasty and control subjects will be compared by logistic regression with covariate adjustment. The data will be presented by total numbers of events and by total numbers of subjects with at least one event. The complication rates including subsequent fractures will be descriptively analyzed through two years post-treatment. The rates and the corresponding 95% exact confidence intervals will be computed.

Based on 12-month plain radiographs, as compared to baseline radiographs, we will compare the proportion of subjects in the two study arms suffering at least one new-onset fracture using the binomial test. We will use the Student t-test to compare the two groups in terms of the mean number of new fractures and the mean number of vertebral levels between the treated levels and new fracture levels.

Non-serious adverse events will be tabulated individually and overall through one year post treatment. The rates will be estimated for the device-treated and control subjects separately and the exact binomial 95% confidence limits will be calculated.

### Safety Monitoring

If at any time after the procedure (vertebroplasty or control) is performed there are any changes in physical health, the event(s) will be reported to the appropriate authorities (DSMB, NIH, and all site PIs and IRBs) as soon as it is known and will be followed until resolution.

An external six-member Data Safety Monitoring Board (DSMB) is monitoring the study in accordance with NIAMS guidelines. The DSMB determined appropriate stopping criteria and will review the data after 73 and 90 subjects have one-month Roland and pain outcomes for evidence of treatment efficacy or unusual rates of adverse outcomes. KIA Research, Inc. manages the safety monitoring for NIAMS, and serious adverse events will be reported directly to NIAMS and KIA Research, Inc., who will distribute this information to the DSMB chair and safety officer, as they occur. Subsequent vertebral fractures (adjacent and non-adjacent) will be a reportable event, not an adverse event. The principal investigator will notify each site IRB of any death regardless of where the death occurred.

### Sample Size

In unpublished pilot data on 88 patients, we found an 8.6 point average reduction on the Roland (standard deviation of 6.7) at 4 weeks following the vertebroplasty procedure. We originally planned to enroll 249 subjects but had to adjust our expectations to 166 subjects due to difficulty with enrolment. With at least 90% follow-up at one month, we expect to have 75 subjects in each arm (150 total) assessed. This sample size yields 95% power to detect a 4-point effect size, and maintains good power for a 3-point effect size (78% power).

We will use a 0–10 numerical pain rating scale to test the hypothesis that short and long-term pain relief is greater in the percutaneous vertebroplasty group than in the control group. In a pilot study, we found a mean reduction of 7.1 (2.7 SD) points on this pain rating scale in a group of patients who received vertebroplasty [24]. A sample size of 150 has greater than 95% power to detect an effect size of 1.75 and a greater than 80% power to detect a 1.25-point effect size.

### Data Management

Data acquisition, handling, and storage are under the direction of the senior programmer of the Data Coordinating Center at the University of Washington. An HTML form-based web application is in use for conducting subject assessments as an efficient method for managing data for this multi-center, national trial. Data will be housed in a secure, centralized database at the University of Washington. The system will automatically perform edit and logic checks to validate the data for quality and completeness as they are being entered. A quality control audit will be performed on a monthly basis and the results printed in an interim report. Staff at the data-coordinating center will monitor the compliance of each study hospital with the study procedures and data quality.

We will document the number and proportion of subjects eligible for and compliant with each follow-up. Careful efforts will be made to minimize missing data, though some will inevitably occur. Subjects who withdraw from the study will be tabulated with the reasons for the withdrawal. Subjects who die during the study (unrelated to study or procedure) will be tabulated also. If the proportion of subjects withdrawn is larger than the expected 15% at one month and additional 10% at one year from either arm, an analysis of the demographic and prognostic characteristics will be made between subjects who withdraw and those who remain in the study. For continuous variables, parametric or non-parametric analysis of variance will be used. For categorical variables, Chi-square or Fisher's exact test will be applied.

When individual items are missing from a scale, we will calculate the percent of missing items. If the missing items are less than 10%, we will impute values using the mean of the remaining items. If more than 10%, the scale score will be missing, and unavailable for analysis at that time point.

## Discussion

Although extensively utilized throughout North America for palliation of pain, vertebroplasty still has not undergone rigorous study. The study outlined above represents the first randomized, controlled study that can account for a placebo effect in the setting of vertebroplasty.

Waning enrolment caused the addition of foreign sites to the study to have the number of subjects needed for an acceptable sample. Approval was obtained from the State Department and the NIH and some foreign sites were initiated into the study and enrolment increased. We now anticipate enrolling a total of 100 subjects. Assuming a 90% follow up rate at one month, we maintain good power (80%) to detect group differences in Roland of 4.0 or more and 87% power for differences in pain of at least 1.75.

Results from this study should provide compelling evidence as to whether or not internal stabilization of non-healing, painful, osteoporotic vertebral compression fractures offers clinical benefit.

## Conclusion

Achieving the sample size of enrolled participants will be difficult especially as the eligibility criteria are so limiting and most potential vertebroplasty patients come from quite a distance and are not willing to return at one month and one year.

## Competing interests

The author(s) declare that they have no competing interests.

## Authors' contributions

DK and JJ were responsible for identifying the research question and contributing to drafting of the study protocol. BC, PH, WH, LS, JT, and LG have all contributed to the development of the protocol and study design, as members of the research team. LG and DK were responsible for the drafting of this paper, although all authors provided comments on the drafts and have read and approved the final version.

## Pre-publication history

The pre-publication history for this paper can be accessed here:


